# Impact of anti-smoking advertising on health-risk knowledge and quit attempts across 6 European countries from the EUREST-PLUS ITC Europe Survey

**DOI:** 10.18332/tid/96251

**Published:** 2018-12-17

**Authors:** Sarah O. Nogueira, Ann McNeill, Marcela Fu, Christina N. Kyriakos, Ute Mons, Esteve Fernández, Witold A. Zatoński, Antigona C. Trofor, Tibor Demjén, Yannis Tountas, Krzysztof Przewoźniak, Anne C. K. Quah, Geoffrey T. Fong, Sara C. Hitchman, Constantine I. Vardavas, on behalf of the EUREST-PLUS consortium

**Affiliations:** 1 Catalan Institute of Oncology (ICO), Bellvitge Biomedical Research Institute (IDIBELL), L’Hospitalet de Llobregat, Barcelona, Spain; 2 University of Barcelona, Barcelona, Spain; 3 King’s College London (KCL), London, United Kingdom; 4 UK Centre for Tobacco and Alcohol Studies, King’s College London (KCL), London, United Kingdom; 5 European Network on Smoking and Tobacco Prevention (ENSP), Brussels, Belgium; 6 University of Crete (UoC), Heraklion, Greece; 7 Cancer Prevention Unit and WHO Collaborating Centre for Tobacco Control, German Cancer Research Center (DKFZ), Heidelberg, Germany; 8 Health Promotion Foundation (HPF), Warsaw, Poland; 9 European Observatory of Health Inequalities, The President Stanisław Wojciechowski State University of Applied Sciences, Kalisz, Poland; 10 University of Medicine and Pharmacy ‘Grigore T. Popa’ Iasi, Iasi, Romania; 11 Aer Pur Romania, Bucharest, Romania; 12 Smoking or Health Hungarian Foundation (SHHF), Budapest, Hungary; 13 University of Athens (UoA), Athens, Greece; 14 Maria Skłodowska-Curie Institute-Oncology Center (MSCI), Warsaw, Poland; 15 University of Waterloo (UW), Waterloo, Canada; 16 Ontario Institute for Cancer Research, Toronto, Canada; 17 National Addiction Centre, King’s College London, London, United Kingdom

**Keywords:** knowledge, Europe, smokers, anti-smoking advertising, quit attempts

## Abstract

**INTRODUCTION:**

Exposure to anti-smoking advertising and its effects differ across countries. This study examines the reported exposure to anti-smoking advertising among smokers and its relation to knowledge of smoking harms and quit attempts in six European countries.

**METHODS:**

Data come from Wave 1 of the International Tobacco Control (ITC) 6 European Country (6E) Survey (Germany, Greece, Hungary, Poland, Romania, Spain) carried out among smokers between June and September 2016 (n=6011). Key measures included whether participants had noticed anti-smoking advertising in the last six months in 6 different channels, their knowledge of 13 adverse smoking/second-hand smoking health effects and if they had made at least one quit attempt in the last 12 months. Multivariate logistic regression models were used in the analysis.

**RESULTS:**

Across the six countries, only 35.2% of smokers reported being exposed to any anti-smoking advertising. Television was the most common channel identified (25.7%), followed by newspapers and magazines (13.8%), while social media were the least reported (9.5%). Participants 18–24 years old were significantly more likely to have noticed advertisements on the Internet than participants >55 years old (24.3% vs 4.9%; OR=5.15). Participants exposed to anti-smoking advertising in all six channels were twice more likely to have a higher knowledge of smoking risks than those not exposed (2.4% vs 97.6%, respectively; OR=2.49). The likelihood of making a quit attempt was increased by 10% for each additional channel through which smokers were exposed to anti-smoking advertising.

**CONCLUSIONS:**

Knowledge of health risks of smoking tended to be higher in countries that aired a campaign in recent years. Exposure to anti-smoking advertising, in the six channels combined, was related to higher smoking knowledge of risks and to more quit attempts. Future anti-smoking mass media campaigns should consider advertising in all dissemination channels to increase the awareness of the dangers of smoking.

## INTRODUCTION

Globally, more than 7 million people die each year due to tobacco-related diseases^1^. In all, 86% are the result of direct tobacco use, while about 13% are the result of non-smokers being exposed to second-hand smoke^1^. This epidemic is one of the worst ever for humanity, and ironically entirely preventable. The continent with the highest prevalence of tobacco smoking among adults is Europe, with around 28% of the population smoking regularly in 2015^1^. The World Health Organization (WHO) has estimated that tobacco use is currently responsible for 16% of adult (over 30 years old) deaths, with many of these occurring prematurely^1^.

Education, communication and public awareness of tobacco-related harms are key objectives of the WHO Framework Convention on Tobacco Control (FCTC), the first global public health treaty aiming to tackle the tobacco epidemic^2^. The countries that signed the treaty are committed to develop and implement a series of evidence-based tobacco control measures to reduce the demand for tobacco.

Article 12 of the WHO FCTC requires Parties to promote and strengthen education and public awareness of the harms of tobacco^2^. To assist parties to meet these obligations, WHO developed guidelines and resources to facilitate the implementation of WHO FCTC, such as the MPOWER strategy that covers six evidence-based measures for effective tobacco control^3^. One of the measures proposed by MPOWER is anti-smoking mass media campaigns (ASMMCs). In general, ASMMCs expose large audiences to anti-smoking messaging via outdoor channels, such as billboards and posters, printed media like newspapers and magazines, social media and the internet, but the most commonly used channels for ASMMC are television and radio^4^. ASMMCs, in these platforms, spread their message to large audiences repeatedly during a defined period, for relatively low cost per person reaching large populations more quickly and efficiently than other communication programmes^5^.

In Europe, countries are at different stages in the development and implementation of policies regarding ASMMCs. According to WHO, Romania, Germany and Poland ran anti-smoking campaigns between 2014 and 2016^6–8^. While these countries have implemented ASMMCs in recent years, to date these campaigns have not been evaluated in terms of their efficacy on changing smoking behaviours or on promoting social awareness of smoking harms. Therefore, the present study aims to describe the reported exposure to anti-smoking advertising in six different channels across six European countries. Moreover, we explore whether exposure to anti-smoking advertising is related to knowledge of smoking health risks and quit attempts, with consideration of sociodemographic variables.

## METHODS

### Study design

The cross-sectional data used come from Wave 1 of The International Tobacco Control Policy Evaluation Project (ITC) 6 European Country (6E) Survey within the EUREST-PLUS Project, a Horizon2020-funded project, which aims to evaluate the impact of the European Union (EU) Tobacco Products Directive (TPD) and WHO FCTC implementation in the EU^9-11^. The ITC 6E Survey is a longitudinal cohort study conducted in Germany (DE), Greece (GR), Hungary (HU), Poland (PL), Romania (RO), and Spain (ES). The fieldwork was conducted between June and September 2016^11^. The overall survey sample comprised 6011 nationally representative adult cigarette smokers, aged 18 years and older, that had smoked more than 100 cigarettes in their lives (about 1000 participants in each country of the study). Sampling followed the Eurobarometer Survey sampling design^12^ and was done using geographical strata determined by Nomenclature of Territorial Units for Statistics (NUTS) regions crossed with the degree of urbanisation (urban, intermediate, rural). Approximately 100 area clusters were sampled in each country, with the aim of recruiting 10 adult smokers per cluster. Clusters were allocated to strata proportionally with 18-years-and-older population size. Within each cluster, household addresses were sampled using a random walk design. One randomly selected male smoker and one randomly selected female smoker were chosen for interview from a sampled household, where possible. Screening of households continued until the required number of smokers from the cluster had been interviewed. All interviews were conducted face-to-face by interviewers using computer-assisted personal interviewing^11^. Further details on the ITC 6E Survey methodology and response rates are available elsewhere^11,13^.

### Ethics

The study protocol was approved by the Office of Research Ethics at the University of Waterloo (Canada), and by the corresponding ethics committees of each participant country. The EUREST-PLUS Project is registered in Clinicaltrials.gov (registration number NCT02773836).

### Measures

#### Demographics and smoking status

Sociodemographic characteristics studied were: country, sex, age (18–24, 25–39, 40–54, and 55 years and older), education (low, medium, high), household income (low, medium, high), level of urbanisation (urban, intermediate, rural), and smoking status (daily, weekly, monthly smoker).

#### Noticing anti-smoking advertising

Participants were asked: ‘Now I would like you to think about advertising or information that talks about the danger of smoking, or encourages quitting. In the last 6 months, how often have you noticed such advertising or information?’. Response options were ‘never’, ‘rarely’, ‘sometimes’, ‘often’, ‘very often’, ‘refused’ and ‘don’t know’. Those participants who answered ‘rarely’, ‘sometimes’, ‘often’ or ‘very often’, were asked: ‘In the last 6 months, have you noticed advertising or information that talks about the dangers of smoking, or encourages quitting, in any of the following 6 places: On television? On the radio? In newspapers or magazines? On posters or billboards? On the internet? On social media?’. Response options were ‘yes’, ‘no’, ‘don’t know’, and ‘refused’ to each place. ‘Refused’ answers were excluded from the analyses, and the remaining answers were dichotomised as ‘yes’ versus ‘otherwise’.

The answers for each of the 6 questions were summed to produce a composite index score (range: 0–6) of exposure to anti-smoking advertising, referred to as the Anti-Smoking Advertising Index (AAI). Those who answered ‘never’ or ‘don’t know’ to the filter question were also included in the AAI composite and assigned a score of zero, i.e. did not notice any anti-smoking advertising in any of the 6 channels.

#### Knowledge of smoking health risks

Respondents were also asked: ‘Based on what you know or believe, does smoking cause: Impotence? Lung cancer? Blindness? Mouth cancer? Throat cancer? Stroke? Emphysema? Bronchitis? Tuberculosis? Heart attack on smokers? Lung cancer on second-hand smokers? Heart attack on second-hand smokers? Asthma in children from second-hand smoke?’. Response options were ‘yes’, ‘no’, ‘don’t know’, and ‘refused’. Those who refused to answer were excluded from the analyses. The remaining responses were dichotomised as ‘yes’ versus ‘otherwise’. The Risk Knowledge Index (RKI) was created by summing the number of ‘yes’ responses across the 13 diseases/health effects (range: 0–13)^14^. Thus, the higher the RKI score, the greater the respondents’ awareness of the diseases caused by smoking and second-hand smoke.

The distribution of the RKI score was highly right-skewed; therefore, the scale was recoded into a dichotomous item by median split (median=10), as no/low (score <10) and high (score ≥ 10), for use as a dichotomous outcome variable, as previously done^15^.

#### Quit attempts

Respondents were asked: ‘Have you made an attempt to quit smoking in the last 12 months?’. Response options were ‘yes’, ‘no’, ‘don’t know’, and ‘refused’. ‘Refused’ answers were excluded from the analyses. The other responses were dichotomised as ‘yes’ versus ‘otherwise’.

### Analysis

Data from all six countries were combined into one dataset. Initially, data were collected from 6011 respondents; however, a total of 206 participants were sequentially excluded from the analyses of the study as a consequence of incomplete data for the following: education (37 respondents), noticing anti-smoking advertising (17 respondents), questions used in construction of the RKI (145 respondents), and attempts to quit (7 respondents).

Multivariate logistic regression was used to examine the association between: a) each of the six countries and noticing anti-smoking advertising in the six channels, b) the RKI and the AAI, and c) quit attempts in the last 12 months and the AAI. All regression models were adjusted for sociodemographics and smoking status. All analyses incorporated weights derived from the complex sampling design.

## RESULTS

### Noticing anti-smoking advertising

In total, 35.2% of the respondents reported noticing anti-smoking advertising in the previous six months in at least one channel, while only 2.4% reported being exposed to all six channels. Table 1 presents the percentage and the adjusted odds ratio (AOR) of self-reported exposure to anti-smoking advertising across the six channels by sociodemographics and smoking status. Television was the channel where most smokers noticed anti-smoking advertising (25.7%), with participants in RO (46.8%; AOR=4.22, 95% CI: 2.75–6.48) and PL (28.6%; AOR=2.15, 95% CI: 1.38–3.36) being significantly more likely to report exposure to anti-smoking advertising on television than those in HU (15.8%). Radio had the second lowest overall percentage of participants noticing anti-smoking advertising (10.5%), with significant differences in exposure to the radio between participants in RO (19.8%; AOR=2.90, 95% CI: 1.71–4.94) and HU (6.3%). Regarding anti-smoking advertising on posters and billboards (13.1% of the sample exposed), participants in RO (20.7%; AOR=2.62, 95% CI: 1.61–4.26) and in DE (20.0%; AOR=2.51, 95% CI :1.50–4.20) were significantly more likely to notice them than those in HU (7.7%). Participants in DE (21.7%; AOR=2.67, 95% CI: 1.59-4.48) and RO (18.3%; AOR=2.25, 95% CI: 1.36–3.74) were significantly more likely to notice anti-smoking advertising in newspapers and magazines than those in HU (8.5%).

**Table 1:** Percentages and odds ratios of self-reported exposure to anti-smoking advertising among smokers from 6 European Countries in 2016, N=5805

	*Television*		*Radio*		*Posters and billboards*		*Newspapers and magazines*		*Internet*		*Social media*	
	*%*	*AOR (95% CI)*	*%*	*AOR (95% CI)*	*%*	*AOR (95% CI)*	*%*	*AOR (95% CI)*	*%*	*AOR (95% CI)*	*%*	*AOR (95% CI)*
**Country**												
Hungary	15.8	1.00	6.3	1.00	7.7	1.00	8.5	1.00	8.2	1.00	6.7	1.00
Germany	17.7	1.05 (0.65–1.67)	6.8	0.89 (0.50–1.58)	20.0	2.51 (1.50–4.20)	21.7	2.67 (1.59–4.48)	12.3	1.49 (0.85–2.60)	8.9	1.25 (0.68–2.29)
Greece	26.7	1.62 (1.02–2.59)	8.9	1.02 (1.26–1.88)	11.3	1.17 (0.68–2.02)	14.0	1.39 (0.81–2.40)	15.1	1.53 (0.90–2.61)	9.4	1.16 (0.66–2.03)
Poland	28.6	2.15 (1.38–3.36)	14.3	2.24 (1.26–3.99)	12.1	1.31 (0.75–2.28)	13.6	1.52 (0.87–2.68)	12.4	1.38 (0.78–2.43)	8.5	1.13 (0.62–2.07)
Romania	46.8	4.22 (2.75–6.48)	19.8	2.90 (1.71–4.94)	20.7	2.62 (1.61–4.26)	18.3	2.25 (1.36–3.74)	21.4	2.77 (1.66–4.61)	17.2	2.58 (1.52–4.39)
Spain	18.9	1.10 (0.71–1.70)	7.0	0.94 (0.51–1.74)	6.6	0.62 (0.34–1.16)	6.6	0.60 (0.34–1.07)	8.7	0.85 (0.48–1.50)	6.5	0.74 (0.42–1.32)
**Sex**												
Male	26.5	1.00	11.2	1.00	13.4	1.00	14.5	1.00	13.7	1.00	10.3	1.00
Female	24.9	0.95 (0.85–1.09)	9.6	0.90 (0.74–1.09)	12.7	0.98 (0.82–1.16)	13.1	0.88 (0.74–1.04)	12.2	0.90 (0.75–1.07)	8.7	0.88 (0.71–1.09)
**Age** (years)												
18–24	30.3	1.00	10.8	1.00	17.9	1.00	14.3	1.00	24.3	1.00	20.0	1.00
25–39	24.9	0.90 (0.67–1.20)	11.0	1.17 (0.79–1.75)	14.5	0.87 (0.63–1.21)	14.7	1.15 (0.82–1.60)	17.5	0.74 (0.55–0.99)	12.7	0.65 (0.47–0.90)
40–54	26.9	1.09 (0.83–1.45)	10.5	1.19 (0.81–1.74)	13.0	0.74 (0.53–1.03)	14.2	1.12 (0.79–1.59)	13.1	0.52 (0.38–0.70)	8.9	0.41 (0.29–0.57)
55+	23.9	0.91 (0.68–1.22)	9.8	1.17 (0.80–1.73)	10.3	0.59 (0.40–0.84)	12.3	1.01 (0.72–1.42)	4.9	0.19 (0.13–0.29)	3.9	0.19 (0.12–0.31)
**Education**												
Low	21.8	1.00	7.5	1.00	11.0	1.00	11.7	1.00	7.5	1.00	6.0	1.00
Medium	28.2	1.10 (0.89–1.34)	12.3	1.43 (1.06–1.94)	14.2	1.23 (0.97–1.56)	15.1	1.21 (0.95–1.53)	15.1	1.84 (1.45–2.32)	11.2	1.73 (1.29–2.32)
High	27.4	1.00 (0.74–1.36)	11.7	1.28 (0.83–1.97)	14.7	1.20 (0.84–1.70)	14.8	1.18 (0.84–1.66)	21.4	2.39 (1.70–3.34)	13.7	1.91 (1.31–2.79)
**Income**												
Low	27.7	1.00	11.1	1.00	13.3	1.00	15.0	1.00	11.1	1.00	8.6	1.00
Medium	28.6	0.89 (0.73–1.89)	12.5	0.97 (0.72–1.30)	14.5	0.97 (0.74–1.27)	14.4	0.83 (0.64–1.06)	13.4	0.86 (0.65–1.14)	10.1	0.85 (0.63–1.16)
High	26.7	0.86 (0.66–1.13)	10.7	0.83 (0.57–1.23)	16.1	1.00 (0.72–1.39)	17.1	1.00 (0.71–1.41)	19.1	1.25 (0.92–1.70)	13.9	1.14 (0.80–1.64)
No answer	18.4	0.71 (0.54–0.95)	6.4	0.65 (0.44–0.95)	8.2	0.80 (0.56–1.15)	9.3	0.86 (0.60–1.21)	9.5	0.90 (0.63–1.30)	6.2	0.74 (0.47–1.16)
**Urbanisation**												
Urban	24.0	1.00	11	1.00	13.8	1.00	15.1	1.00	13.8	1.00	10.3	1.00
Intermediate	25.5	1.15 (0.87–1.53)	10.0	1.01 (0.72–1.42)	12.1	0.87 (0.62–1.21)	14.4	0.99 (0.71–1.36)	12.6	1.02 (0.74–1.40)	8.8	0.91 (0.63–1.33)
Rural	28.3	0.99 (0.74–1.31)	10.5	0.86 (0.60–1.23)	13.4	0.93 (0.65–1.34)	12.2	0.82 (0.58–1.17)	12.4	0.92 (0.65–1.30)	9.6	0.90 (0.63–1.29)
**Smoking status**												
Daily	25.4	1.00	10.2	1.00	12.6	1.00	13.2	1.00	12.7	1.00	9.3	1.00
Weekly	33.8	1.69 (1.17–2.44)	16.4	1.87 (1.08–3.24)	24.4	1.53 (1.02–2.29)	27.6	2.05 (1.40–3.00)	19.6	1.21 (0.82–1.78)	15.1	1.24 (0.78–1.97)
Monthly	30.8	1.94 (0.96–3.91)	15.4	2.04 (0.80–5.22)	15.4	1.08 (0.47–2.50)	23.1	1.87 (0.83–4.18)	17.3	1.53 (0.59–4.00)	13.5	1.73 (0.57–5.24)
**Overall sample**	25.7		10.5		13.1		13.8		13.0		9.5	

Percentages are unadjusted. Multi-level logistic regression adjusted for country, sex, age, education, income, urbanisation, and smoking status; AOR: Adjusted Odds Ratio; CI: Confidence Interval.

The percentage of exposure of the overall sample on the internet was of 13.0%. Participants in RO (21.4%; AOR=2.77, 95% CI: 1.66–4.61) were significantly more likely to notice anti-smoking advertising on the internet than those in HU (8.2%). Social media had the lowest percentage of participants reporting noticing the anti-smoking advertising (9.5%); in this channel, participants in RO had the highest percentage of exposure (17.2%; AOR=2.58, 95% CI: 1.52–4.39) and participants in HU the lowest (6.7%). No significant differences were found in exposure to anti-smoking advertising between participants in HU and ES across any of the 6 channels (Table 1).

In all media, males noticed anti-smoking advertising more frequently, but the differences according to sex were not statistically significant. Those who reported smoking weekly were significantly more likely to report being exposed to anti-smoking advertising on television (33.8%; AOR=1.69, 95% CI: 1.17–2.44), radio (16.4%; AOR=1.87, 95% CI: 1.08–3.24), posters and billboards (24.4%; AOR=1.53, 95% CI: 1.02–2.29), and newspapers and magazines (27.6%; AOR=2.05, 95% CI: 1.40–3.00) than those who smoked daily (25.4%, 10.2%, 12.6% and 13.2%, respectively). Smokers aged 55 years and over were significantly less likely than those younger than 25 years to have noticed anti-smoking advertising on posters and billboards (Table 1). Besides, the older the age group of a participant, the smaller the likelihood of reporting being exposed to advertising via the internet and social media. A similar gradient with education and exposure on social media was observed, with those with more years of education being more exposed. No association was found between any of the 6 channels by sex, income, and urbanisation.

### Knowledge of health risks and the association between Anti-Smoking Advertising Index and Risk Knowledge Index

The mean score of smokers on the RKI was 8.9 (out of 13). Table 2 shows the results of the multivariate association between RKI (low <10 versus high ≥10) and AAI. No significant association was found between the AAI, as a continuous construct, and the RKI. However, those with a score of 6 on the AAI (the highest exposure to anti-smoking advertising), were significantly more likely to have a higher RKI score than those scoring zero on AAI (AOR=2.49, 95% CI: 1.50–4.15).

**Table 2:** Association of Anti-Smoking Advertising Index and Risk Knowledge Index, among smokers from 6 European countries in 2016, N=5805

*Predictors*	*Risk Knowledge Index*	
	*Mean (SD)*	*AOR (95% CI)*
**Country**		
Hungary	8.0 (4.2)	Ref.
Germany	8.3 (3.6)	0.96 (0.65–1.41)
Greece	9.4 (3.1)	1.55 (1.06–2.27)
Poland	9.1 (3.8)	1.46 (0.99–2.15)
Romania	10.0 (3.7)	2.59 (1.77–3.79)
Spain	8.7 (3.4)	1.19 (0.80–1.76)
**Sex**		
Male	8.8 (3.8)	Ref.
Female	9.1 (3.6)	1.17 (1.05–1.30)
**Age (years)**		
18–24	8.4 (3.7)	Ref.
25–39	8.8 (3.8)	1.39 (1.07–1.80)
40–54	9.2 (3.6)	1.68 (1.31–2.15)
55+	9.0 (3.7)	1.60 (1.23–2.09)
**Education**		
Low	8.5 (3.9)	Ref.
Medium	9.2 (3.6)	1.23 (1.02–1.47)
High	9.3 (3.3)	1.15 (0.90–1.47)
**Income**		
Low	8.9 (3.8)	Ref.
Medium	9.2 (3.6)	1.11 (0.91–1.37)
High	9.0 (3.6)	1.03 (0.80–1.33)
No answer	8.5 (3.8)	0.92 (0.71–1.19)
**Urbanisation**		
Urban	8.9 (3.6)	Ref.
Intermediate	9.1 (3.6)	1.04 (0.81–1.35)
Rural	8.8 (4.0)	0.96 (0.73–1.27)
**Smoking status**		
Daily	8.9 (3.7)	Ref.
Weekly	9.3 (3.5)	1.15 (0.79–1.67)
Monthly	9.7 (2.8)	1.13 (0.61–2.09)
**AAI**		
(OR for 1 unit increase in score)	–	1.04 (0.99–1.10)

Multi-level logistic regression adjusted for country, sex, age, education, income, urbanisation, and smoking status; AOR: Adjusted Odds Ratio; CI: Confidence Interval; AAI: Anti-Smoking Advertising Index; Ref.: Reference category.

### Association between the Anti-Smoking Advertising Index and quit attempts

In all, 18% of the sample reported to have made a quit attempt within the previous 12 months. Table 3 presents the association between exposure to anti-smoking advertising and quit attempts in the last 12 months. There was an overall significant positive effect (AOR=1.10, 95% CI: 1.03–1.17) in the association of these variables — the more channels a participant was exposed to, the more likely she/he would have made a quit attempt; for each additional channel a smoker was exposed to, the likelihood of making a quit attempt increased by 10%.

**Table 3:** Association of Anti-Smoking Advertising and quit attempts in the last 12 months, among smokers in six European Countries in 2016, N=5805

*Predictors*	*Reported quit attempts in the last 12 months*	
	*%*	*AOR (95% CI)*
**Country**		
Hungary	11.1	Ref.
Germany	18.8	1.22 (0.76–1.95)
Greece	15.5	0.85 (0.51–1.41)
Poland	18	1.25 (0.78–1.98)
Romania	27	2.07 (1.34–3.18)
Spain	17.2	1.24 (0.76–2.03)
**Sex**		
Male	17.5	Ref.
Female	18.5	1.07 (0.91–1.25)
**Age (years)**		
18–24	23.8	Ref.
25–39	19.9	0.86 (0.63–1.16)
40–54	15.4	0.64 (0.48–0.85)
55+	17.3	0.72 (0.54–0.96)
**Education**		
Low	16.2	Ref.
Medium	18.3	1.01 (0.83–1.25)
High	22.6	1.43 (1.04–1.95)
**Income**		
Low	17.8	Ref.
Medium	19.4	1.05 (0.86–1.29)
High	17.6	0.88 (0.66–1.15)
No answer	16	1.01 (0.76–1.33)
**Urbanisation**		
Urban	18.5	Ref.
Intermediate	17.4	1.10 (0.88–1.37)
Rural	18.1	0.99 (0.78–1.26)
**Smoking status**		
Daily	17.3	Ref.
Weekly	32.4	1.85 (1.26–2.73)
Monthly	26.9	1.58 (0.76–3.29)
**AAI**		
OR for 1 unit increase in score)	_	1.10 (1.03–1.17)

Multi-level logistic regression adjusted for country, sex, age, education, income, urbanisation, and smoking status; AOR: Adjusted Odds Ratio; CI: Confidence Interval; AAI: Anti-Smoking Advertising Index; Ref.: Reference category.

## DISCUSSION

The overall findings from this study indicate a significant association between a high AAI score and having made a quit attempt in the last 12 months. This suggests that anti-smoking advertising may have had a positive impact on smokers, with the result of increasing quit attempts, consistent with previous findings^16^. Additionally, the association of exposure to anti-smoking advertising with quit attempts may be partly explained by the fact that the AAI score was also positively associated with higher smoking risk knowledge (RKI score), but only among respondents who were exposed to all six channels. Furthermore, participants from RO, one of the countries that invested in at least one medium quality ASMMC according to WHO standards^17^ between 2014 and 2016^7^, had significantly higher odds of reporting exposure to anti-smoking advertising (AAI score) than participants in countries where no national anti-smoking campaign took place.

While knowledge of smoking risks was expected to be a mediator between exposure to anti-smoking advertising and quit attempts, our data show that only participants exposed to all the six channels had a higher knowledge of smoking risks. This could point to other possible mediators of the association between exposure to anti-smoking messaging and quit attempts. For example, possibly these campaigns denormalise smoking and make people more likely to make a quit attempt, or they could generate emotional reactions resulting in smokers being more inclined to make quit attempts^18^. We used the whole sample as a denominator and not only those using the channels, thus our measure of exposure captured two effects: the true exposure and the media channel use, i.e. the exposure conditional to use. This might produce an underestimation of the true effect, and should be considered when developing public health interventions.

Television (watched on a TV set or via the Internet) is the preferred channel among those residing in Europe, with 84% viewing it every day or almost every day^19^. This is a possible reason why television is the channel that a higher proportion of participants reported being exposed to anti-smoking advertising. This finding corroborates evidence that anti-smoking advertisements on television are twice as likely to be remembered than those on radio^20–22^.

The Internet and social media have a lower reach to the European population than television: 59% of the Europeans use the Internet, and 35% of them use online social networks every day or almost every day^19^. These relatively new media channels, to date, have attracted little research attention regarding their use as tools to improve awareness of tobacco harms, being used mostly for tobacco cessation initiatives^23^.

Young adults and those completing higher levels of education had significantly higher odds of being exposed to anti-smoking advertising on the Internet and social media than older participants and those with lower education level. This population group is also the one that uses the internet and social media the most^19^, while having the lowest knowledge of smoking risks. Therefore, future anti-smoking campaigns, in these channels, should focus on the young and highly educated adults regarding the outcomes and impacts intended to be achieved.

Even though DE, PL and RO ran medium quality ASMMCs between 2014 and 2016, according to WHO^6–8,24–26^, there were significant differences in all outcome measures evaluated between participants in these countries; with participants in RO reporting significantly higher odds of noticing anti-smoking advertising, of quit attempts, and of smoking risk knowledge, than participants in DE and PL.

DE was an exception among the countries that ran a campaign in recent years. Although WHO reported that there was a national ASMMC between 2014 and 2016, participants in DE had the lowest odds ratio of being exposed to anti-smoking advertising on television and radio, among all six countries. A possible explanation for these findings is that the ASMMC reported by WHO in DE was not a population-wide campaign but rather a specific campaign. Although most countries that execute ASMMC do not repeat the effort frequently (most gaps are longer than 2 years)^17^, research has shown that the withdrawal of an ASMMC is associated with a decline in beneficial effects, pointing to the need for a more persistent effort in this area^4,27–29^. Another possible explanation for these findings is that the ASMMC reported by WHO in DE happened in 2014 and did not extend to 2016, the time frame of the question related to exposure to anti-smoking advertising in this study.

Cross-country differences may be related to the content of the messages from these campaigns. Evidence shows that anti-smoking advertisements that generate high levels of negative emotion are associated with greater recall rates and impact on smoking attitudes and intentions^15,18^. Another hypothesis for these discrepancies is related to differences in campaign characteristics, such as duration and intensity, which may influence the effectiveness in changing smoking behaviours^5,30^.

Participants in GR, HU and ES reported being exposed to anti-smoking information and advertising even though there was no public investment in national ASMMCs in these countries between 2014 and 2016. This may be due to private initiatives, regional or international anti-smoking campaigns and/or media news coverage related to tobacco control policy implemented and tobacco control research publicised in these countries. Such private initiatives could also explain that even though the national campaign in PL did not include television and radio advertising, many participants were exposed to anti-smoking advertising on these channels. For example, in PL a significant amount of money was used by pharmaceuticals to advertise smoking cessation drugs on prime-time TV and on radio channels, which might mimic anti-smoking advertising^31^. This additional information related to tobacco control policies may increase the effectiveness of messages from ASMMC, as reported in a review^30^.

This study has some limitations. First, self-reported measures are susceptible to recall bias, especially over periods of 6 months^32^. Advertising needs to be remembered to be reported faithfully; those that are salient and more recent are more likely to be remembered and reported correctly^32^. Anti-smoking advertising made within the past 3 months is related to a higher likelihood of making a quit attempt during that time^16^. Second, our exposure measurement was also not very specific to ASMMCs, as the question comprised anti-tobacco advertising and information, which for instance included anti-smoking news coverage. Moreover, the measure of exposure to anti-smoking advertising did not assess the intensity and frequency of these events, only whether they occurred, and this may have contributed to the lack of effect on smoking risk knowledge. Third, the questions used to construct the RKI had simple yes/no answer options and regarded exclusively diseases associated with smoking. Fourth, the question used to assess quit attempts did not specify what the interviewee should consider as a quit attempt, which might bias the results, as reported in the literature^33^. Additionally, evidence points to the association between quit attempts and motivation to quit^34^, however, this variable was not included in our model. Fifth, cross-sectional survey data were used in this study. This methodology precludes any inference to be made about the direction of causality. Thus, it is possible that adult smokers who are more inclined to make a quit attempt may be more likely to notice, recall and report being exposed to anti-smoking advertising. Sixth, participants were asked about the occurrence of quit attempts in the last 12 months, while they were asked about being exposed to anti-smoking advertising in the last 6 months. Therefore, these questions covered different periods. This might represent a limitation, as smokers may have tried to quit smoking before being exposed to any anti-smoking advertising. Finally, those who decided not to disclose their income were a group that had a significantly lower association with exposure to anti-smoking advertising, in our analysis. Although controlling for education, urbanisation, and sex, may diminish the influence of these missing data, this might bias the results.

On the other hand, this study has strengths. First, this is the first study to investigate the reach of anti-smoking campaigns and messages across these 6 European countries. Additionally, it provides updated data on the level of smoking risk knowledge across a large and representative sample of smokers in these 6 countries. Also, some implications for policymakers can be derived. Television seems to be the channel that most smokers are exposed to and recall seeing anti-smoking advertising the most; therefore, future anti-smoking mass media campaigns should focus on this channel. In addition, it may be necessary that these campaigns occur more frequently, and over a more extended period, to ensure that the benefits generated by them do not decline over time. Future longitudinal research should explore changes in anti-smoking advertising campaigns in these countries and analyse the nuances that result in differences in noticing anti-smoking advertising. Further investigation of the use of the internet and social media as tools to reach the young is needed.

## CONCLUSIONS

Exposure to anti-smoking advertising is associated with smoking quit attempts. Such exposure was also correlated with smokers’ knowledge of smoking risks. Those exposed to anti-smoking advertising in the six channels studied were approximately 2.5 times more likely to have a higher knowledge of smoking risks than those not exposed. This indicates that future anti-smoking mass media campaigns should consider advertising in all dissemination channels to increase the awareness of the dangers of smoking.
